# Genetically engineering encapsulin protein cage nanoparticle as a SCC-7 cell targeting optical nanoprobe

**DOI:** 10.1186/2055-7124-18-21

**Published:** 2014-12-23

**Authors:** Hyojin Moon, Jisu Lee, Hansol Kim, Somin Heo, Junseon Min, Sebyung Kang

**Affiliations:** Department of Biological Sciences, School of Life Sciences, Ulsan National Institute of Science and Technology (UNIST), Ulsan, 689-798 South Korea

**Keywords:** Encapsulin, Protein cage nanoparticle, Cell targeting, Nanoprobe, Delivery

## Abstract

**Background:**

Protein cage nanoparticles are promising nanoplatform candidates for efficient delivery systems of diagnostics and/or therapeutics because of their uniform size and structure as well as high biocompatibility and biodegradability. Encapsulin protein cage nanoparticle is used to develop a cell-specific targeting optical nanoprobe.

**Results:**

FcBPs are genetically inserted and successfully displayed on the surface of encapsulin to form FcBP-encapsulin. Selectively binding of FcBP-encapsulin to SCC-7 is visualized with fluorescent microscopy.

**Conclusions:**

Encapsulin protein cage nanoparticle is robust enough to maintain their structure at high temperature and easily acquires multifunctions on demand through the combination of genetic and chemical modifications.

## Background

Conventional drugs and diagnostic probes tend to diffuse rapidly and get distributed throughout the body easily upon systematic administration [1]. Non-targeted treatments generally cause detrimental side effects in normal cells and tissues and the reduction of the effectiveness of the treatment [2]. The targeted delivery of diagnostic or/and therapeutic reagents to desired sites is a challenging, but promising, task for the early diagnosis of diseases as well as effective and localized treatment of diseases.

A variety of inorganic or organic nanoparticles, including metal nanoparticles [3, 4], micelles [5, 6], polymers [7–9], liposomes [9–11], and protein cages [1, 12], have been investigated as efficient delivery vehicles for diagnostic or/and therapeutic reagents because they have great potential to improve the pharmacological properties of drugs and maximize the localized treatment of diseases [13]. The manipulation of size and surface of nanoparticles increase their accessibility to disease sites and circulation time in the bloodstream due to their enhanced permeability and retention (EPR) effect; this improves the biodistribution of drugs [14, 15].

Protein cages, such as ferritins [12, 16–19], viral capsids [17, 20–24], lumazine synthase [20, 25, 26], and encapsulin [27], are promising nanoplatform candidates for efficient delivery systems of diagnostics and/or therapeutics because they have uniform size and structure as well as high biocompatibility and biodegradability [1]. Protein cages are spontaneously self-assembled from multiple copies of one or a few types of protein subunits in a precisely controlled manner. In addition, they can be manipulated genetically and chemically to have a desired function, using a rational design based on atomic resolution structural information.

Encapsulin, a novel protein cage nanoparticle isolated from thermophile *Thermotoga maritima*, is assembled from 60 copies of identical 31 kDa monomers having a thin and icosahedral *T =* 1 symmetric cage structure with interior and exterior diameters of 20 and 24 nm, respectively [28]. Although the exact function of encapsulin in *T. maritima* is not clearly understood yet, its crystal structure has been recently solved and its function was postulated as a cellular compartment that encapsulates proteins such as DyP (Dye decolorizing peroxidase) and Flp (Ferritin like protein) which are involved in oxidative stress responses [28, 29]. This study implied that encapsulin has a large enough central cavity (20 nm in inner diameter) to encapsulate a large amount of therapeutic and/or diagnostic reagents and we recently constructed a heat stable encapsulin variant through genetic engineering and demonstrated the utility of engineered encapsulin as a versatile drug delivery nanoplatform.

In the present study, we genetically engineered a novel protein cage nanoparticle, encapsulin, to display cell-specific targeting peptides (DCAWHLGELVWCT) onto the surface in a controlled manner and demonstrated its selective binding to Squamous cell carcinoma (SCC-7) cell line exclusively.

## Methods

### Genetic modification of encapsulin and protein cage purification

We started with a genetically modified encapsulin, which has only one cysteine per subunit at position 123. Cell targeting peptide with linker (GGGGGGDCAWHLGELVWCTGGGGG) was inserted into residues between 138 and 139 of encapsulin by an established polymerase chain reaction (PCR) protocol using pET-30b based plasmids containing genes encoding encapsulin [20].

Peptide insertion was confirmed by DNA sequencing and confirmed DNAs were transformed into the competent *E.coli* strain BL21 (DE3) and the protein cages were over-expressed in *E.coli*. The pelleted *E.coli* cells from 1.0 L of culture were resuspended in 35 mL of phosphate buffer (50 mM sodium phosphate and 100 mM sodium chloride, pH 6.5). Lysozyme was added and the solution was incubated for 30 min at 4°C. The suspension was sonicated for 10 min in 30 s intervals, and subsequently centrifuged at 12000 g for 1 hr at 4°C. Encapsulin protein cage was purified by size exclusion chromatography (SEC) after heat precipitation for 10 min at 65°C [20].

### Quartz crystal microbalance (QCM) measurements

QCM experiments were performed using Q-Sense E4 and standard gold QCM sensors (Q-Sense, Sweden) as described previously [30]. Briefly, the system was operated in flow mode with a pump and temperature was maintained at 25.0 ± 0.1°C. Each sample solution was introduced to the measurement chamber with a pump and continuously measured for 3 min prior to the subsequent introductions. Protein cages and rabbit IgGs were introduced at concentrations of approximately 100 μg/ml and 50 μg/ml, respectively, in phosphate buffer (50 mM phosphate, 100 mM NaCl, pH 6.5). Resonance frequencies were measured simultaneously at seven harmonics (5, 15, 25, 35, 45, 55 and 65 MHz). For clarity, only the normalized frequency of the third overtone is shown.

### Surface plasmon resonance (SPR) analysis

SPR experiments were performed with carboxyl dextran CM-5 gold chips on a Biacore 3000 device (Biacore AB, Sweden) at 25 ^o^C using a PBS buffer as a running solution. Rabbit IgG was coupled to the surface of a CM-5 sensor chip by standard amine-coupling chemistry on the SPR instrument as described previously, with slight modifications [31]. Briefly, a mixture of EDC (0.4 M) and NHS (0.6 M) was injected onto the chip at a flow rate of 10 μl/min to activate carboxyl groups on the sensor surface and subsequently 20 μg/ml of rabbit IgG was added at the same flow rate for 7 min. Excess reactive groups were blocked with 1 M ethanolamine (pH 8.0). Encapsulin capture by rabbit IgG was examined by applying various amounts (1, 5, 10, 25, 50, and 100 nM) of encapsulin (PBS, pH 7.4) to the surface at a flow rate of 30 μl/min.

### Mass spectrometry of modified encapsulin protein cage

For ESI-TOF analysis, encapsulin protein cages were loaded onto the MassPREP Micro-desalting column (Waters) and eluted with a gradient of 5-95% (v/v) acetonitrile containing 0.1% formic acid at a flow rate of 500 μL/min. The molecular masses of each species can be determined from the charges and the observed mass-to-charge (m/z) ratio values. Mass spectra were acquired in the range of m/z 500-3000 and deconvoluted using MaxEnt1 from MassLynx version 4.1 to obtain the average mass from multiple charge state distributions [20].

### Cell culture and confocal fluorescence microscopy

All cell lines in this article were obtained from the Korean cell line bank (KCLB) and maintained in a humidified atmosphere of 5% CO_2_ and 95% air at 37°C. SCC-7 cells were incubated in RPMI 1640 medium with 10% fetal bovine serum (FBS), 1% penicillin/streptomycin. MDA-MB-231 and HepG2 cells were incubated in RPMI 1640 medium with 10% FBS, 1% streptomycin. KB cells were cultured in RPMI1640 medium with L-glutamine (300 mg/L), 10% FBS, 1% streptomycin, 25 mM HEPES and 25 mM NaHCO_3_. Hela cells were incubated in DMEM medium with 4.5 g/L glucoseand L-glutamine, 10% FBS and 1% streptomycin. Cells were grown on microscope cover glasses (18 mm Ø) in 12-well culture plate (SPL, 30012). The cells were fixed with 4% paraformaldehyde in PBS and washed 2 times with PBS containing 0.1% Tween 20. The fixed cells were blocked with 5% BSA, 5% FBS, and 0.5% Tween 20 in PBS at 4°C for 18 hr and blocking buffer was aspirated. Encapsulin and FcBP-encapsulin were treated for 20 hr at 4°C. Before sealing, the cells on the cover glasses were washed 3 times (15 min) and nuclei were stained with DAPI. Images of stained substrates were collected using Olympus Fluoview FV1000 confocal microscope (Olympus, UOBC).

## Results and discussions

### Construction of FcBP-presenting encapsulin protein cage nanoparticles

**Figure 1:**
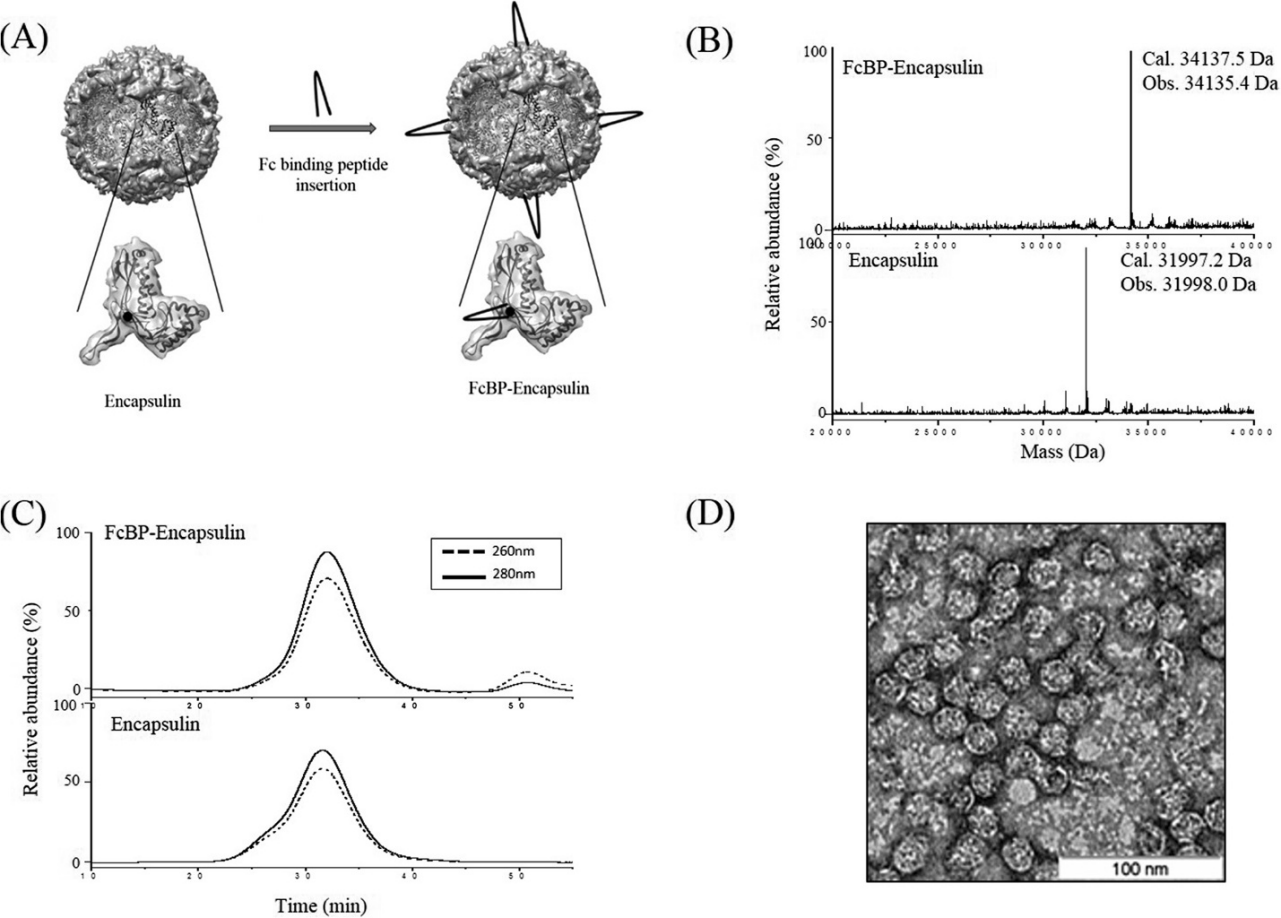
**Characterization of the FcBP-Encapsulin. (A)** Surface and ribbon diagram representations of encapsulin (PDB: 3DKT). The position to introduce the FcBP is indicated with black dot. The FcBP is represented as v-shaped line. **(B)** Molecular mass measurements of dissociated subunits of encapsulin (bottom) and FcBP-encapsulin (top). Calculated and observed molecular masses were indicated. **(C)** Size exclusion profiles of encapsulin (bottom) and FcBP-encapsulin (top). **(D)** Transmission electron microscopic image of negatively stained FcBP-encapsulin with 2% uranyl acetate.

To use encapsulin protein cage as a nanoplatform for a targeted delivery vehicle, we genetically introduced a short peptide (GGGGGGDCAWHLGELVWCTGGGGG) with extra glycine residues onto the surface loop region of encapsulin (Figure 1A). This short peptide is known to have a high affinity to the Fc region of immunoglobulin G (IgG) originated from rabbit [32] and therefore it is called Fc-binding peptide (FcBP). Based on the crystal structure of encapsulin, the loop regions are exposed on the surface and easily form intra-strand disulfide bonds (underlines in amino acid sequence) due to their proximal distant induced by the loop configuration (Figure 1A) [33]. FcBP insertion was confirmed by DNA sequencing and molecular mass measurement of subunit of FcBP inserted encapsulin protein cages (FcBP-encapsulin).

Purified FcBP-encapsulin was characterized by mass spectrometry (MS), size exclusion chromatography (SEC), and transmission electron microscopy (TEM). Each FcBP-encapsulin subunit exhibited a molecular mass of 34,135.4 Da, which is well matched with the predicted molecular mass of 34,137.5 Da (Figure 1B, top panel). FcBP-encapsulin eluted at the same position as encapsulin on SEC, suggesting that FcBP-encapsulin forms a stable 60-mer cage architecture (Figure 1C). TEM images of stained FcBP-encapsulin protein cages confirmed an intact cage architecture with a uniform size distribution with a diameter of approximately 25 nm (Figure 1D). These results indicate that FcBP-encapsulin forms an intact protein cage architecture without significant changes in size, composition, and architecture.

### Inserted FcBPs are displayed on the surface of encapsulin

**Figure 2:**
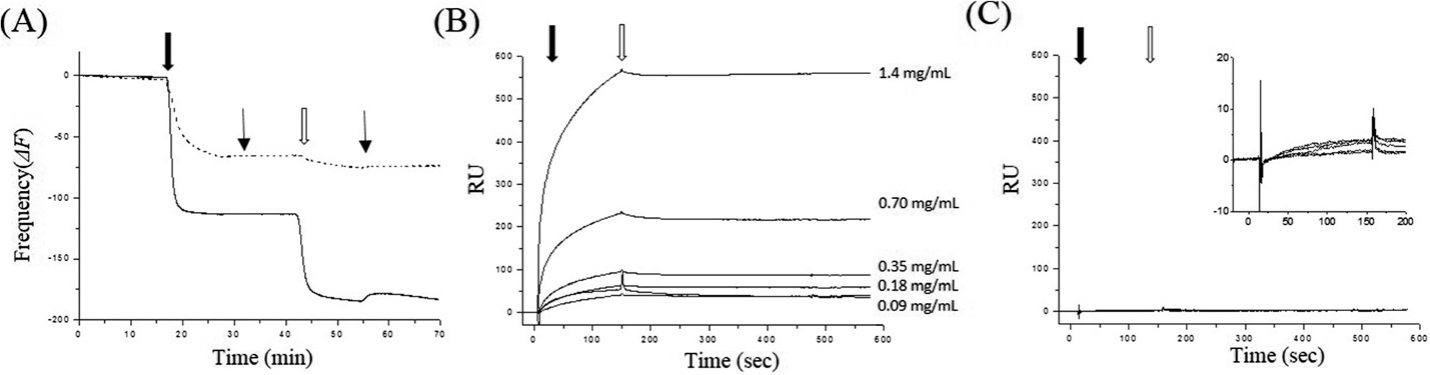
**Binding behaviors of FcBP-Encapsulin to antibodies. (A)** QCM resonance frequency change (-Δ*F*) profiles of either encapsulin (dashed line) or FcBP-encapsulin (solid line) on the gold QCM sensors and subsequent deposition of rabbit IgG on the monolayer of FcBP-encapsulin. Filled and open arrows indicate the timing of encapsulin variant and rabbit IgG introductions, respectively. Thin arrows indicate the timing of buffer washing. SPR analyses of FcBP-encapsulin **(B)** and encapsulin **(C)** bindings to rabbit IgG immobilized gold SPR sensors. Filled and open arrows indicate the timing of encapsulin variant introductions and buffer washing, respectively. Inset of **(**C**)** is amplified graph of low RU ranges.

A synthetic cyclic FcBP (D*C*AWHLGELVW*C*T) exhibited a high affinity to rabbit IgGs with the value of *K*_*d*_ =305 nM [32]. In order to test whether inserted FcBPs are on the surface of encapsulin, we performed real-time quartz crystal microbalance (QCM) analysis. Typically, deposition of molecules on the QCM sensor results in decreases in resonance frequency (-Δ*F*) and the extent of frequency changes are sensitive to the masses of the deposited molecules [30, 34]. We have shown that QCM analysis is a nice method to determine protein-protein interactions. Various types of protein cages, including ferritins, lumazine synthase, and virus-like particles (VLP), showed strong binding capability to the gold QCM sensor and form uniform monolayers without any surface modifications [30, 34]. The resonance frequency value of FcBP-encapsulin (Figure 2A, solid line, filled arrow) itself was lower than that of encapsulin (Figure 2A, dashed line, filled arrow) only probably due to the increase in mass of the FcBP insertions to encapsulin (24 amino acids, an approximate 8% increase in mass) (Figure 2A). The resonance frequency of each sample decreased, plateauing when it reached a certain value. In addition, no additional changes were observed even with continuous introduction of further sample and subsequent washing, indicating that the encapsulin had formed a uniform monolayer regardless of the FcBP insertion and that there was negligible amount of non-specific absorption (Figure 2A, thin arrow). Subsequently, we introduced rabbit IgG solution over the encapsulin- or FcBP-encapsulin-monolayered QCM sensors and measured frequency changes in real-time (Figure 2A). While the frequency of FcBP-encapsulin-monolayered QCM sensor decreased dramatically upon introduction of rabbit IgG, the frequency of encapsulin-monolayered QCM sensor remained unchanged (Figure 2A, open arrow). Extensive washing removed only slight amounts of non-specifically associated rabbit IgG with most of the initially bound rabbit IgG remaining bound (Figure 2A, thin arrow). These results indicated that the inserted FcBPs are well displayed on the surface of encapsulin and they are fully accessible for Fc regions of rabbit IgGs to bind. These data also imply that exterior displayed FcBPs can be used as ligands for target selective delivery of FcBP-encapsulin.

Next, we examine whether FcBP-encapsulin can recognize the immobilized targets and selectively bind to them. To do that, we performed surface plasmon resonance (SPR) analysis. In contrast to QCM studies, we first immobilized rabbit IgGs on the surface of an SPR CM-5 sensor chip [32] and introduced either FcBP-encapsulin or encapsulin at several concentrations. If the inserted FcBPs are exposed on the surface of encapsulin and accessible to the biomolecules, they will selectively bind to the immobilized rabbit IgGs resulting in gradual increases in SPR responses depending on the amounts of introduced FcBP-encapsulin or encapsulin. As we expected, gradual increases in SPR responses (RU) were observed upon introduction of FcBP-encapsulin (Figure 2B, filled arrow), with RU values reaching a plateau at each concentration (Figure 2B, open arrow). Consistent with previous QCM results, apparent dissociation of FcBP-encapsulin from the immobilized rabbit IgG was not observed even after extensive buffer washing (Figure 2B). However, encapsulin did not bind to the immobilized rabbit IgGs at all (Figure 2C and inset) regardless of the amount of introduced encapsulin. These results suggest that the FcBPs displayed on the encapsulin can recognize the immobilized target, rabbit IgG, and allow to selectively bind to them and multiple FcBPs on the surface of FcBP-encapsulin may cooperatively capture the immobilized rabbit IgGs resulting in extremely strong binding (Figure 2B).

### Specific binding of FcBP-encapsulin to the SCC-7 cells

FcBP-encapsulin has only one cysteine per subunit at position 123 (60 cysteines per cage) which is known to be chemically active. To attach fluorescent probes to the FcBP-encapsulin, we treated FcBP-encapsulin with activated fluorescein-5-maleimide (F5M). F5M-conjugated FcBP-encapsulin (fFcBP-encapsulin) was separated from unreacted F5M by SEC, and we confirmed that every subunit is labeled with one F5M using MS (data not shown).

**Figure 3:**
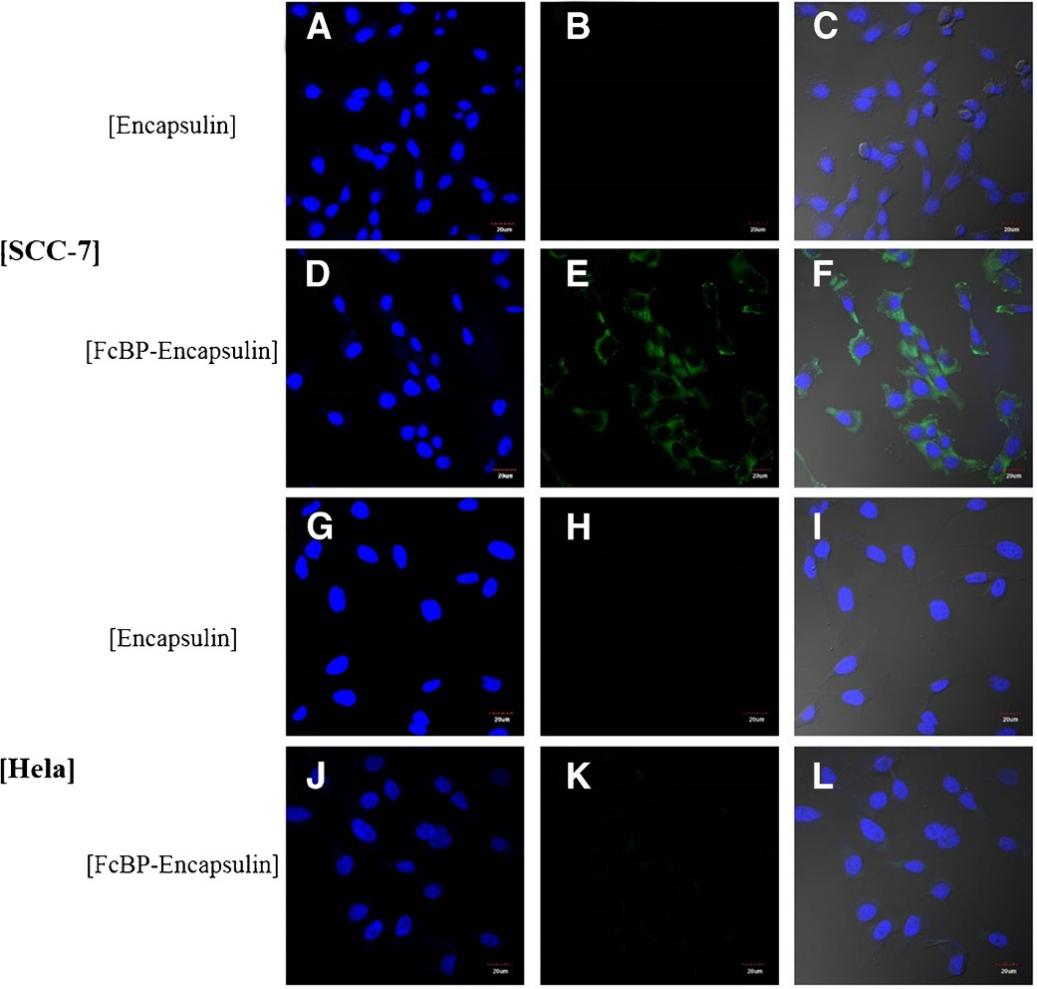
**Fluorescent microscopic images.** Fluorescent microscopic images of SCC-7 cells **(A-** **F)** treated with f-encapsulin **(A-** **C)** and fFcBP-encapsulin **(D-** **F)**. Fluorescent microscopic images of Hela cells **(G-** **L)** treated with f-encapsulin **(G-** **I)** and fFcBP-encapsulin **(J-** **L)**. DAPI (left rows), fluorescein (middle rows), and merged (right rows) were represented.

**Figure 4:**
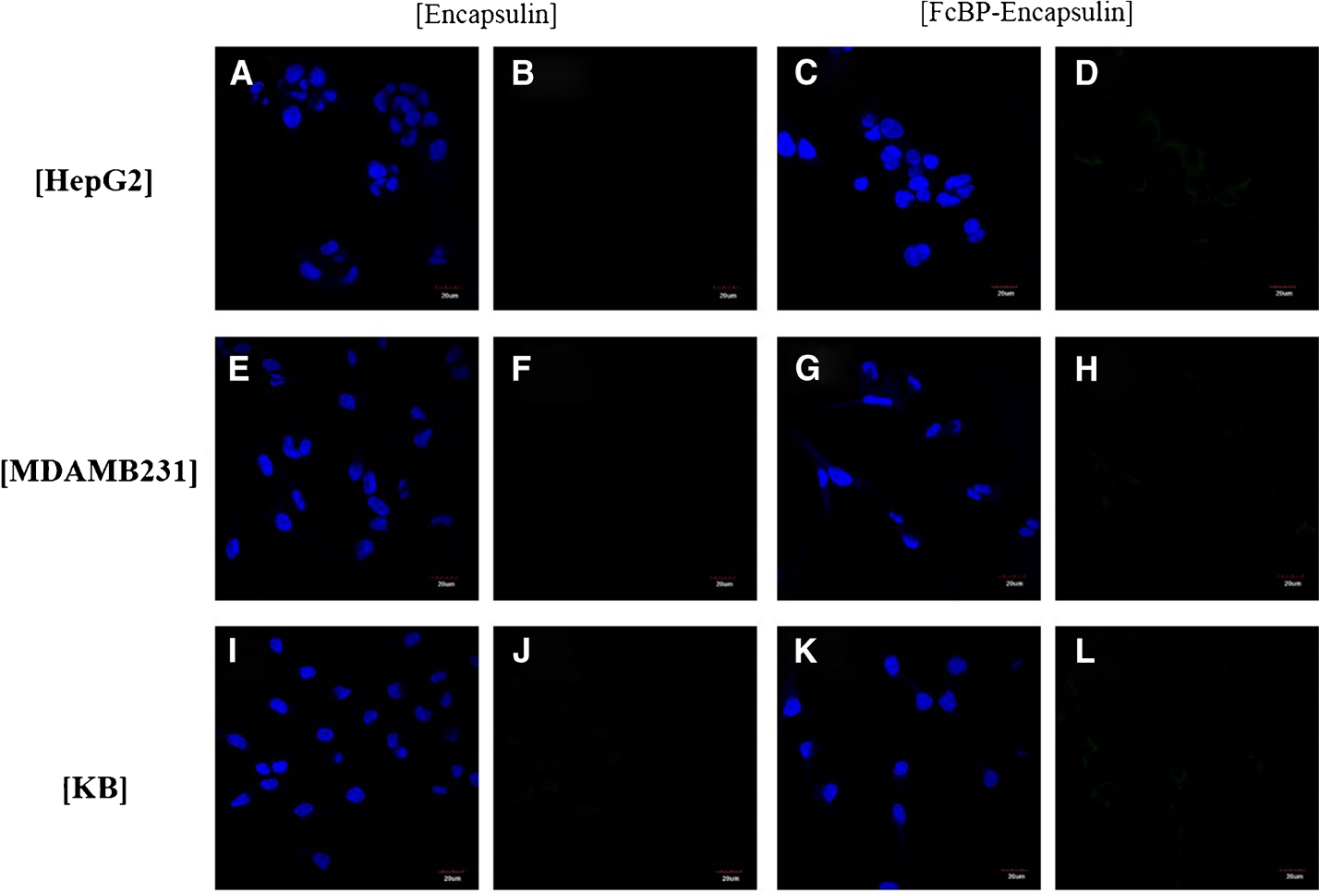
**Fluorescent microscopic images.** Fluorescent microscopic images of HepG2 cells **(A,** **D)** treated with f-encapsulin **(A,** **B)** and fFcBP-encapsulin **(C,** **D)**. Fluorescent microscopic images of MDA-MB-231 cells **(E,** **H)** treated with f-encapsulin **(E,** **F)** and fFcBP-encapsulin **(G,** **H)**. Fluorescent microscopic images of KB cells **(I,** **L)** treated with f-encapsulin **(I,** **J)** and fFcBP-encapsulin **(K,** **L)**. DAPI **(A, C, E, G, I, K)** and fluorescein **(B, D, F, H, J, L)** were represented.

To determine whether FcBP-encapsulin can bind a particular cell selectively, we treated a variety of cell lines with fFcBP-encapsulin and visualized them with fluorescent microscopy. F5M-conjugated encapsulin (f-encapsulin) was also treated in parallel as control. We first tried SCC-7 cells which overexpress a cell surface glycoprotein CD44 involved in cell-cell interactions, cell adhesion and migration. While f-encapsulin did not bind to SCC-7 cells at all (Figure 3A-C), fFcBP-encapsulin bound to SCC-7 cells (Figure 3D-F). Fluorescence images of SCC-7 treated with fFcBP-encapsulin showed cytosolic accumulation of fFcBP-encapsulin (Figure 3E, F). These data suggest that FcBP-encapsulin recognizes surface markers of SCC-7 cells, selectively binds to them, and is internalized. Furthermore, the pretreatment of SCC-7 cells with anti-CD44 antibody completely blocked the binding of FcBP-encapsulin (data not shown). We then chose Hela cells and treated them with same way of SCC-7 cells. We did not observe any binding of neither fFcBP-encapsulin nor f-encapsulin to Hela cells (Figure 3G-L). To investigate cell selectivity of FcBP-encapsulin further, we prepared HepG2 hepatocyte cells, MDA-MB-231 breast cancer cells, and KB epithelial cells additionally and treated them with fEcBP-encapsulin or f-encapsulin. None of cell lines we prepared showed the evidence of specific binding of either fEcBP-encapsulin or f-encapsulin (Figure 4). Although through studies about surface molecules that FcBP-encapsulin binds to should be done, these data demonstrate that FcBP-encapsulin selectively recognizes and binds to SCC-7 cells and has a potential to be used as an efficient molecular imaging probe *in vitro*. FcBP-encapsulin has a large internal cavity (20 nm in diameter) and it can be used for encapsulating chemicals, nanomaterials, and proteins. Thus, encapsulin has a potential to be used as a modular template for developing a versatile, multifunctional theranostic system, which has specific cell targeting ligands and diagnostic and therapeutic reagents simultaneously

## Conclusions

In this study, we engineered encapsulin protein cage nanoparticle as a SCC-7 cell targeting optical nanoprobe. Fc-binding peptide with linker (GGGGGGD*C*AWHLGELVW*C*TGGGGG, FcBP) was introduced onto the surface loop region of encapsulin. Insertion of FcBPs and integrity of FcBP-encapsulin were confirmed by various biophysical methods, including MS, SEC, and TEM. QCM and SPR analyses demonstrated that FcBP-encapsulin indeed polyvalently displayed FcBPs on the surface of encapsulin by monitoring specific binding of it to rabbit IgG. Fluorescently labeled FcBP-encapsulin selectively bound to the SCC-7, but not to the Hela, HepG2, MDA-MB-231 or KB cells as shown by fluorescence imaging. Since FcBP-encapsulin is robust and acquire multifunctions on demand, it can be used as a nanoplatform for developing a multifunctional theranostic system.
